# Updating Traumatic Brain Injury Classification for Surgeons: Integrating the CBI‐M Framework Into Trauma and Acute Care Practice

**DOI:** 10.1002/wjs.70440

**Published:** 2026-06-02

**Authors:** Ruben Peralta, Santiago Cardona‐Collazos, Ali Msheik, Wendy D. Gonzalez, Andrés Salazar Restrepo, Laura M. Loaiza‐Cardona, Andres M. Rubiano

**Affiliations:** ^1^ Trauma Surgery Hamad Medical Corporation Doha Qatar; ^2^ Department of Surgery Universidad Nacional Pedro Henríquez Urena School of Medicine Santo Domingo Dominican Republic; ^3^ MEDITECH Foundation Cali Colombia; ^4^ Neurosurgery Neuroscience Institute Hamad Medical Corporation Doha Qatar; ^5^ Neuroscience Institute Neurotrauma Group El Bosque University Bogotá Colombia

**Keywords:** biomarkers, classification, glasgow coma scale, imaging, personalized medicine, traumatic brain injuries

## Abstract

**Background:**

Traumatic brain injury (TBI) remains a major cause of death and disability worldwide. Traditional classification based on the Glasgow Coma Scale (GCS) provides a shared clinical language but insufficiently captures the biological heterogeneity, imaging variability, and contextual modifiers that influence outcomes. In January 2024, the National Institute of Neurological Disorders and Stroke (NINDS) convened a multidisciplinary working group to propose the Clinical–Biomarkers–Imaging–Modifiers (CBI‐M) framework. This review aims to summarize the rationale, structure, and potential clinical relevance of the CBI‐M framework for trauma and acute care surgeons.

**Methods:**

A narrative literature review was conducted using PubMed and Scopus databases with the terms “traumatic brain injury,” “classification,” “biomarkers,” “neuroimaging,” and “personalized medicine.” Articles published between 2000 and 2024 were prioritized, with emphasis on consensus statements, multicenter cohort studies (e.g., TRACK‐TBI, CENTER‐TBI), and materials from the NINDS TBI Classification and Nomenclature Workshop. Reference lists were manually screened to identify additional relevant publications. The review synthesizes conceptual foundations, domain structure, and practical implementation considerations of the CBI‐M model.

**Results:**

The CBI‐M framework introduces a multidimensional and dynamic approach to TBI characterization by integrating four domains: Clinical assessment (detailed GCS components, pupillary reactivity, post‐traumatic amnesia, and structured symptom documentation); Biomarkers, including GFAP, UCH‐L1, S100 B, NfL, and pTau, which provide objective measures of neuronal and astroglial injury with defined temporal kinetics; Imaging, emphasizing standardized CT terminology within the first 24 h and harmonized radiologic lexicons; and Modifiers, incorporating psychosocial, environmental, and comorbidity factors that influence outcomes. This integrated model may support more comprehensive characterization of TBI, facilitate interdisciplinary communication, and enable structured documentation across trauma systems.

**Conclusions:**

The CBI‐M framework represents a conceptual shift from severity‐based classification toward a multidomain approach to TBI characterization. For trauma surgeons, it offers a structured framework that may support more comprehensive documentation and facilitate integration with registry‐based quality improvement and translational research. At present, its role is best understood as an evolving model with potential for future clinical applicability, pending prospective validation and assessment of feasibility across diverse trauma systems.

## Graphical abstract

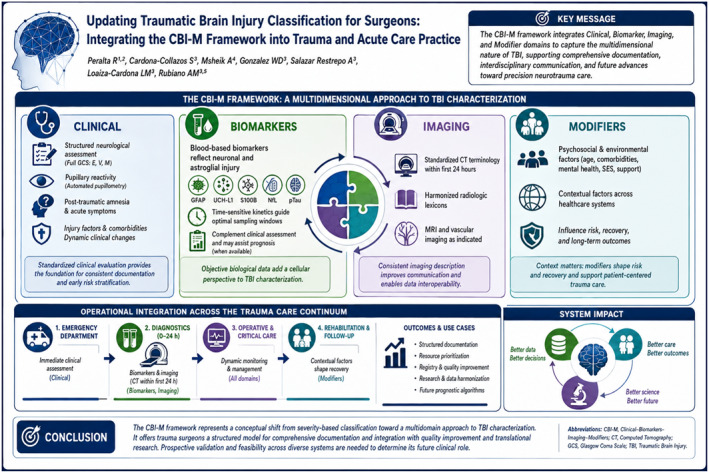

## 1 Introduction

Traumatic brain injury (TBI) remains a critical global health challenge and a leading cause of death and disability, particularly among individuals under 40 years of age [1, 2]. Its incidence continues to rise, accounting for nearly one in every 50 emergency department visits [3]. A central element in TBI management is the classification of injury severity, traditionally based on the Glasgow Coma Scale (GCS) score obtained after initial resuscitation. This system has proven invaluable for establishing a common clinical language and generating reliable prognostic estimates, with severe TBI consistently associated with higher morbidity and mortality [4, 5, 6, 7, 8]. However, as understanding of TBI pathophysiology has increased in the genomics and proteomic era, the limitations of this traditional classification have become evident. The GCS focuses exclusively on clinical presentation, overlooking biological, anatomical, and temporal dimensions that may influence outcomes [9, 10]. Consequently, it may be overly simplistic for a condition as heterogeneous and dynamic as TBI, limiting its capacity to guide management and research under modern standards.

In response, the National Institute of Neurological Disorders and Stroke (NINDS) convened experts in January 2024 to propose a revised framework, known as the Clinical, Biomarkers, Imaging and Modifiers (CBI‐M) framework. This model integrates molecular, anatomical, and clinical domains to capture the multidimensional nature of TBI, with the goal of improving characterization and supporting future advances in prognostic modeling, personalized care, and translational research [3]. This narrative review examines the rationale and implications of this pivotal update, emphasizing its potential relevance for surgeons seeking more comprehensive frameworks to characterize TBI in acute care settings.

## 2 Methods

Relevant literature was identified through PubMed and Scopus using predefined search terms (“traumatic brain injury,” “classification,” “biomarkers,” “neuroimaging,” and “personalized medicine”). Articles published between 2000 and 2024 were considered, with emphasis on consensus statements, multicenter studies (e.g., TRACK‐TBI and CENTER‐TBI), and reports from the NINDS TBI Classification and Nomenclature Workshop. Reference lists of key publications were manually reviewed. As a narrative review, this study did not employ a systematic search strategy, formal study selection process, or risk‐of‐bias assessment, which may limit reproducibility and completeness.

## 3 Results

### 3.1 The Need for a New Classification System

Disease classification integrates clinical knowledge with therapeutic strategies and prognostic expectations, serving as the foundation for research and clinical decision‐making. An ideal system stratifies patients according to relevant pathoanatomic and pathophysiologic mechanisms, guiding both treatment and prognosis [9, 11].

TBI presents a set of unique classification challenges due to its dynamic nature, heterogeneity, and the complexity of brain function. Existing systems typically address only one dimension of the condition, mechanism (penetrating or blunt), pathoanatomic imaging patterns (such as Marshall or Rotterdam scales), or clinical severity via the Glasgow Coma Scale (GCS) [11].

Among these, the GCS‐based severity classification remains the most widely adopted but also the most susceptible to external factors like alcohol, drugs or systemic hypotension. It categorizes TBI as mild (13–15), moderate (9–12), or severe (< 9), with strong interobserver reliability and clear prognostic implications [4, 5, 6, 7, 8]. However, by modern standards, it may provide an incomplete representation of TBI heterogeneity. It does not capture underlying mechanisms, neuroimaging findings, or extracranial injuries, leading to broad and heterogeneous patient groups within each category. Two patients with identical GCS scores may have dramatically different pathologies (primary injuries) and outcomes [9, 11, 12, 13]. The static nature of this system further limits its applicability, it cannot accommodate temporal evolution, pediatric or neurologically impaired patients, and poorly discriminates mild injuries, which constitute over 80% of all cases [14].

Recent multicenter studies such as TRACK‐TBI and CENTER‐TBI, along with advances in molecular and imaging sciences, have expanded understanding of TBI heterogeneity. These data suggest that outcomes are influenced not only by injury biology but also by psychosocial and environmental factors, which may vary substantially across geographic regions and healthcare systems [2, 14, 15, 16, 17, 18, 19, 20, 21]. A modern classification framework should therefore account for this variability and consider feasibility across both high‐income and resource‐limited settings [13, 22, 23].

### 3.2 The New Classification Proposal

The newly proposed CBI‐M model introduces a dynamic and multimodal framework for characterizing TBI based on four domains: Clinical, Biomarkers, Imaging, and Modifiers [10, 13]. (Table 1). Rather than functioning as a traditional classification system with weighted or numerical outputs, it is currently best understood as a structured multidomain framework intended to organize clinically relevant information and support future development of more standardized classification approaches [13, 24, 25, 26, 27, 28].

**TABLE 1:** Summary of recommended clinical descriptors for TBI classification to enhance patient characterization across different care scenarios.

Summary: Full GCS with separate specifications for motor, verbal, and eye components (e.g., GCS 14, E3V5M6). GCS should be recorded at the arrival to the emergency room for optimal prognostic impact and consistency. If a confounder for GCS assessment is present (e.g., alcohol, sedation, intubation), it should be explicitly noted. If a GCS component is untestable, it should be explicitly noted and not scored as “1” (e.g., “V(t)” in an intubated patient). In all patients, but particularly in patients with a GCS ≤ 12, pupillary responses should be recorded: This is best done independently of the GCS. Automated pupillometry should be used whenever possible. In all patients with a GCS verbal score ≥ 4 in the emergency room: Post‐traumatic amnesia (PTA) should be assessed and reported using a validated tool. Acute symptoms should be documented and reported, ideally using standardized tools.
Expanded: The same information as in the summary format, in addition to the following: Injury factors, including: Injury mechanism, impact velocity, and impact mitigation (e.g., seat belts, airbags, helmets, etc). In patients with a GCS verbal score ≤ 4, record if there is a history of loss of consciousness (LoC). Extracranial injuries (especially injuries that would, in isolation, mandate admission even without a TBI). Post‐traumatic amnesia duration, determined by prospective serial assessment with a validated tool assessed in different time points post‐injury. Biopsychosocial‐ecological vulnerabilities: Physical and psychological comorbidities, Listing of relevant therapies (especially treatments that affect hemostasis). Age and frailty (using a clinical frailty assessment scale). Socioeconomic status, educational attainment, and employment status Dynamics of clinical change: Determined by neuroworsening, physiological monitoring, and therapy intensity during the treatment course in hospitalized patients. Determined by symptom severity over the next 2 weeks post‐injury, for patients not admitted to the hospital.

Biomarkers: Blood‐based biomarkers (BBM) provide objective measures of neuronal, astrocytic, and vascular injury, complementing clinical assessment [19, 20]. Among the most studied are GFAP, UCH‐L1, S100 B, NfL, and pTau [21]. However, their clinical utility is influenced by strict temporal kinetics, requiring sampling within defined time windows, and by variability in assay availability. These constraints may limit their applicability in settings with delayed presentation, uncertain injury timing, or restricted laboratory access, particularly in resource‐limited environments.

Each biomarker exhibits unique temporal kinetics, which determine the optimal sampling windows. Incorporating these BBMs into TBI characterization may add a cellular‐level perspective and may assist risk stratification when neurological examination is unreliable, where testing is available and performed within appropriate time windows. Table 2 summarizes some relevant characteristics of these BBMs and the recommendations for various time scenarios as proposed.

**TABLE 2:** Characteristics and recommended clinical use of BBMs in the new traumatic brain injury classification system.

Biomarker	Characteristics	Clinical use by scenario
GFAP	A protein that participates in the production of intermediate filaments of the cellular cytoskeleton of astroglia, it is very specific for nervous tissue. Its blood values rise in the first hours after trauma, peak at 20 h, and are maintained until 72 h, after which they decrease.	Acute (0–24 h): Identifies patients who are likely to have a normal head CT and may reduce the need for neuroimaging; predicts traumatic intracranial lesions not detected on CT; and correlates with mortality and 6–12‐month functional outcomes. A threshold of 65 pg/mL indicates a very low risk of CT‐detectable intracranial injury. The biomarker can be measured as a point‐of‐care test in whole blood or in a central laboratory using a plasma sample. Subacute (1–30 days): Assists in diagnosing TBI, predicting functional outcomes, and detecting radiologic abnormalities.
UCH‐L1	A protein located exclusively in neurons, it is one of the most abundant proteins in the brain (1%–2% of total proteins). Its blood levels begin to rise a few minutes after trauma, but it has a short half‐life, with peak levels at 8 h, from which its level starts to decrease.	Acute (0–24 h): Assists in identifying patients with normal CT scans, distinguishing TBI from controls, and predicting mortality and 6‐month outcomes. A cutoff of 400 pg/mL indicates a very low risk of CT‐detectable intracranial injury. Can be measured as a point‐of‐care test in whole blood or in a central laboratory using a plasma sample.
S100 B	An astrocytic protein regulating signal transduction and cell morphology. Although sensitive for acute TBI, its extracranial sources (adipose tissue, muscle, melanocytes) limit specificity. Its short half‐life requires measurement within 3–6 h post‐injury.	Acute (0–24 h): Identifies patients unlikely to need CT; supports TBI diagnosis. A cutoff of 105 μg/L indicates a very low risk of CT‐detectable intracranial injury. Subacute (1–30 days): Aid in predicting delayed radiologic pathology and mortality.
NfL	A component of the axonal cytoskeleton expressed primarily in subcortical axons whose elevation in blood has been correlated with brain injury.	Subacute (1–30 days): Aids in TBI diagnosis, predicts white matter atrophy and long‐term outcomes (6–12 months). Chronic (> 30 days): potential to aid in predicting post‐TBI cerebral atrophy and reduced microstructural integrity.
pTau	Axonal localization protein that has been studied in subacute and chronic TBI.	Chronic (> 30 days): potential to aid in predicting global functional outcome and persistent symptoms 6–12 months after injury.

Abbreviations: GFAP: Glial Fibrillary Acidic Protein; UCH‐L1: Ubiquitin Carboxy‐terminal Hydrolase L1; S100 B: S100 calcium‐binding protein B; NfL: Neurofilament Light Chain; pTau: Phosphorylated Tau. Adapted from the proposed classification framework by NINDS.

Imaging: The imaging domain promotes the standardized description of pathoanatomic findings using common data elements integrated into electronic medical records (EMRs). The framework primarily focuses on CT imaging within the first 24 h post‐injury, while acknowledging the roles of MRI and vascular modalities in subsequent evaluation [32, 33]. Harmonized terminology facilitates consistency in communication among researchers, clinicians, and patients. To delve deeper into the proposed terminology and its implementation, the reader should refer to the summary of the Imaging Working Group available on the NINDS TBI Classification and Nomenclature Workshop website.

Modifiers: Psychological and environmental factors (PEFs) can influence an individual's risk of experiencing a TBI and their short, medium, and long‐term outcomes. Integrating PEFs into clinical assessment may support a more individualized understanding of patient context and recovery risk. Their inclusion in the new TBI classification highlights the importance of a holistic approach, considering not only the medical aspects but also the patient's psychosocial and environmental contexts [13, 34, 35, 36]. Table 3 summarizes the recommendations for PEFs in the proposed new classification system.

**TABLE 3:** Recommendations for PEFs in the proposed new classification system.

Recommendations to include PEFs for evaluation during patient arrival: When assessing TBI patients, consider their age and developmental status. Do not assume that high GCS scores indicate low‐severity TBI, particularly in older adults, as GCS can underestimate the severity of anatomical injuries in this population. Additionally, consider that various pre‐existing factors, such as intoxication or chronic dementias/psychiatric disorders, may lead to either underestimation or overestimation of TBI severity during initial clinical evaluations. For children suspected of experiencing non‐accidental injury, engage in a multidisciplinary team‐based assessment, as distinguishing these injuries from accidental ones can be particularly challenging. Aim to provide trauma‐informed care that considers the broader emotional and social contexts surrounding injuries and make recommendations to mitigate potential problems such as post‐traumatic stress disorder (PTSD) and repeated exposure to violence. Enhance the ability to deliver culturally informed care by assessing patients in their native languages whenever possible and acknowledging cultural factors that may impact their presentation.
Recommendations to include PEFs for discharge planning and clinical follow‐up: Consider the clinical encounter for TBI as an opportunity to recognize and address psychosocial and environmental barriers to recovery, the risk of repeat injuries, and overall health and well‐being. Recommended actions include: Screening for mental health issues, including problematic substance use, and providing relevant interventions. Identifying and addressing socioeconomic barriers, such as food insecurity, housing, transportation challenges, low social support, and financial concerns, by utilizing available resources and social services. Developing or refining evidence‐based injury prevention programs, including violence interruption initiatives for individuals impacted by assaultive or non‐accidental trauma, strategies to identify and address pediatric and elder abuse, and fall prevention efforts for those at increased risk of falls.

### 3.3 Practical Applications for Trauma Surgeons

In acute trauma care, the CBI‐M framework may provide surgeons with a structured approach to organizing patient information across multiple domains [8, 13, 24, 25, 37]. The Clinical component supports consistent documentation, while the Biomarker and Imaging domains may complement assessment where available [29, 30, 31, 38]. However, it remains uncertain whether integration of these domains into a single framework translates into improved bedside decision‐making or reproducible prognostic classification compared with existing tools [13, 32, 33, 34, 35, 36].

**FIGURE 1:**
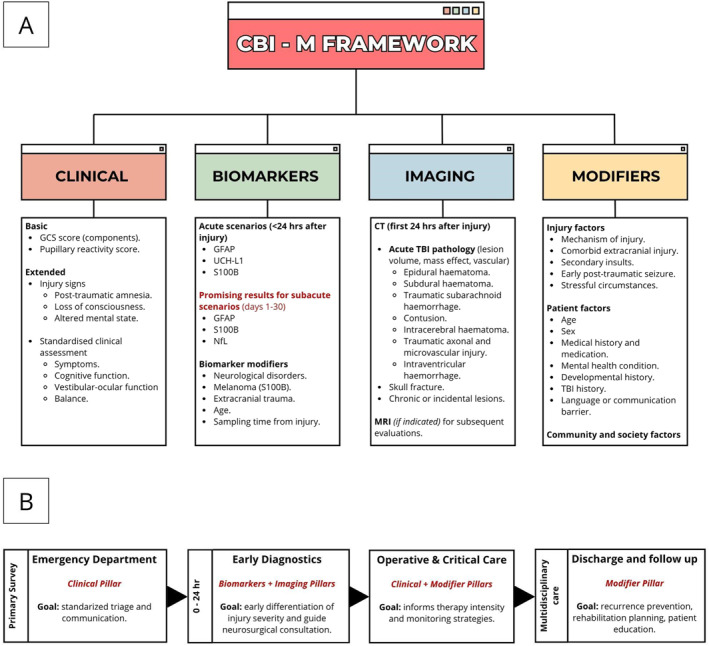
Conceptual and Operational Integration of the CBI‐M Framework for TBI care. (A) Conceptual overview illustrating the four domains of the CBI‐M framework. Each domain captures complementary dimensions of TBI characterization, integrating clinical examination findings, blood‐based biomarkers, standardized imaging lexicons, and contextual modifiers such as age, comorbidities, and psychosocial factors. (B) Operational integration of the CBI‐M framework across trauma care stages. Sequential application of the CBI‐M pillars throughout acute TBI care. Each care phase aligns with its dominant pillar, from structured clinical assessment in the Emergency Department to biomarker and imaging‐guided diagnostics, dynamic monitoring during operative and critical care, and psychosocial modifier‐based rehabilitation planning, linking bedside decision‐making with precision documentation and multidisciplinary coordination.

For trauma surgeons managing polytrauma or mass casualty incidents, structured CBI‐M documentation may assist in organizing clinical information for resource prioritization, integration with triage systems, and registry‐based quality improvement [2, 14, 20, 21]. It may help align bedside documentation with institutional performance metrics and may facilitate future integration with triage and prognostic algorithms (see Figure 1) [13, 20, 22].

At present, there is insufficient comparative evidence to determine whether the CBI‐M framework improves prognostic accuracy beyond established tools such as the Glasgow Coma Scale or CT‐based classifications.

## 4 Discussion

### 4.1 Implementation and Integration

**FIGURE 2:**
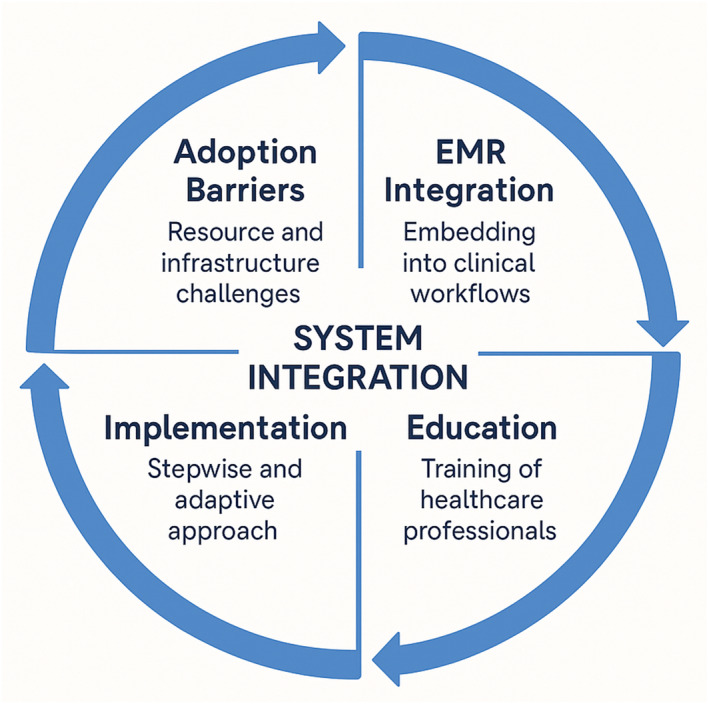
System‐level integration loop of the CBI‐M framework showing adoption, EMR embedding, education, and staged implementation. Cyclic process for integrating the Clinical, Biomarker, Imaging, and Modifier (CBI‐M) framework into trauma systems. The loop connects four interdependent domains, Adoption Barriers, EMR Integration, Education, and Implementation, establishing a continuous improvement cycle that links bedside practice, digital infrastructure, and multidisciplinary training to enable sustainable precision neurotrauma care.

The successful adoption of the CBI‐M framework within trauma systems requires deliberate alignment between clinical workflow, informatics infrastructure, and staff training. Standardizing data acquisition for the Clinical and Modifier domains through structured templates embedded in EMRs ensures reproducibility and facilitates interoperability with trauma registries and research databases [13, 20, 22]. Integration of biomarker assays into emergency laboratory protocols requires consideration of cost, infrastructure, and turnaround time, which may limit implementation outside well‐resourced centers [29, 30, 31, 38]. Advanced imaging lexicons should be harmonized with radiology reporting systems to promote automatic extraction of structured data for analytics and artificial intelligence–assisted prognostication [32, 33]. In parallel, education and competency‐based training of trauma and emergency teams are essential to promote fidelity in framework implementation, particularly in documenting GCS components, pupillary metrics, and psychosocial modifiers [8, 13, 24, 25, 34, 35, 36, 37]. Resource‐limited settings may adopt a tiered implementation approach, prioritizing clinical and modifier domains, while biomarker and advanced imaging components remain aspirational until feasibility is established. Institutional engagement, continuous audit, and feedback mechanisms will be important for sustainability if the framework is to evolve from a theoretical model into a practical tool for quality improvement in trauma care (see Figure 2).

Evidence supporting implementation of the CBI‐M framework in low‐ and middle‐income settings is currently limited, and feasibility may vary depending on access to imaging, laboratory infrastructure, and workforce capacity.

### 4.2 Limitations

While the proposed framework offers a comprehensive conceptual model, it has not yet been validated in prospective clinical studies, and its impact on clinical outcomes, reproducibility, and decision‐making remains uncertain. Additional limitations include variability in access to biomarker assays and advanced imaging, as well as challenges in implementation across heterogeneous healthcare systems. Future research should focus on prospective validation, feasibility assessments, and development of simplified, clinically transferable formats.

## 5 Conclusion

The CBI‐M framework reflects current efforts to develop a more structured multidomain approach to TBI characterization. At present, it should be considered an evolving conceptual model that may support more comprehensive documentation and research standardization. Further prospective validation and feasibility studies are required to determine whether it can develop into a clinically applicable classification or prognostic tool, particularly across diverse healthcare settings. Its current lack of a standardized quantitative scoring system may limit immediate clinical transferability.

## Author Contributions

**Ruben Peralta:** conceptualization, supervision, project administration, writing – review and editing. **Santiago Cardona‐Collazos:** investigation, writing – original draft, data curation. **Ali Msheik:** investigation, writing – original draft, writing – review and editing, project administration. **Wendy D: Gonzalez:** investigation, writing – original draft. **Andrés Salazar Restrepo:** validation, writing – review and editing. **Laura M: Loaiza‐Cardona:** validation, writing – review and editing. **Andres M. Rubiano:** conceptualization, supervision, writing – review and editing. **Originality Statement.** This manuscript has not been previously published, is not under consideration elsewhere, and will not be submitted to another journal while under review. All listed authors meet authorship criteria according to international standards. All authors have participated sufficiently in the work to take public responsibility for the content. The final version of the manuscript has been reviewed and approved by all authors.

## Funding

The authors have nothing to report.

## Ethics Statement

This article does not contain any studies with human participants or animals performed by any of the authors. This manuscript complies with the ethical standards of the institutional and national research committee and with the 1964 Helsinki declaration and its later amendments.

## Conflicts of Interest

The authors declare that they have no competing interests relevant to this work.

## Reporting and Registration

This study was conducted as a narrative review. As such, it did not follow a systematic review methodology and was not prospectively registered in a review registry. Therefore, a PRISMA checklist is not applicable.

## Data Availability

Data sharing not applicable to this article as no datasets were generated or analysed during the current study.
